# Association between venlafaxine use and the risk of withdrawal from nonopioid substances: a nationwide, population-based study

**DOI:** 10.1186/s12954-026-01427-9

**Published:** 2026-03-02

**Authors:** Shyh-Yuh Wei, Brian Meng-Hsun Li, Daniel Hsiang-Te Tsai, Hsuan-Yun Hu, Szu-Yu Lin, Swu-Jane Lin, Chien-Chou Su, Chih-Hsin Pan, Edward Chia-Cheng Lai

**Affiliations:** 1 Department of Psychiatry, National Cheng Kung University Hospital, College of Medicine, National Cheng Kung University, Tainan, Taiwan; 2 School of Pharmacy, Institute of Clinical Pharmacy and Pharmaceutical Sciences, College of Medicine, National Cheng Kung University, Tainan, Taiwan; 3 Population Health Data Center, National Cheng Kung University, Tainan, Taiwan; 4 Institute of Forensic Medicine, Ministry of Justice, New Taipei City, Taiwan; 5 Department of Pharmacy Administration, College of Pharmacy, University of Illinois at Chicago, Chicago, USA; 6 Clinical Innovation and Research Center, National Cheng Kung University Hospital, College of Medicine, National Cheng Kung University, Tainan, Taiwan

**Keywords:** Antidepressants, Case-case-time-control design, Stimulants, Substance use, Venlafaxine, Withdrawal

## Abstract

**Background:**

Appropriate treatments for nonopioid substance use are currently unavailable. Venlafaxine may reduce withdrawal from nonopioid substances, but the effects have not been evaluated. We aimed to investigate the association between venlafaxine use and the risk of withdrawal from nonopioid substances.

**Methods:**

We linked Taiwan’s National Health Insurance Research Database and the Taiwan Illicit Drug Issue Database from January 2012 to December 2019. We used a case-case-time-control study involving a case-crossover analysis and a control-crossover analysis consisting of future cases. The outcomes were withdrawal from substances and all-cause mortality. For each individual, venlafaxine use during the hazard period (day − 8 to − 67 before the outcome) was compared with that during the 60-day reference period (between days − 248 and − 307). Conditional logistic regression was used to determine odds ratios with 95% confidence intervals to evaluate the associations between outcome events and the use of venlafaxine.

**Results:**

The participants’ average age on the index date was 39.5 years (standard deviation 8.7), with 84.1% men and 88.3% having low income. Venlafaxine was significantly associated with a lower risk of withdrawal from substances (odds ratio 0.35, 95% confidence interval 0.13 to 0.96). However, we found no association between the recent use of venlafaxine and all-cause mortality (1.08, 0.55 to 2.14). The point estimates were similar in a series of sensitivity analyses, though not all analyses statistical significance.

**Conclusions:**

This study provides strong ground for clinicians to consider the use of venlafaxine to reduce patient experiencing withdrawal symptoms from substances.

**Supplementary Information:**

The online version contains supplementary material available at 10.1186/s12954-026-01427-9.

## Background

The use of substances is linked to a wide spectrum of medical, psychiatric, and societal challenges for patients, along with notable public disturbances [1]. On a global scale, 1.3% of age-standardized disability-adjusted life-years were attributed to substance use as a risk factor in 2016 [2], with an annual increase exceeding 0.5% [3]. Although there are medications for treating opioid use disorder, despite intensive research in the past decade [4, 5], no pharmacotherapy has yielded conclusive results for the treatment of nonopioid substance use, such as cocaine [6], methamphetamine [7], or cannabis [8].

As such, when clinically treating nonopioid substance use is warranted, physicians often choose medications for managing psychiatric disorders [9]. Venlafaxine elevates synaptic levels of serotonin and norepinephrine and has emerged as a potential treatment option following a positive finding in a clinical trial enrolling individuals with depressive and cocaine use disorders [10]. Furthermore, an animal study also suggested its potential efficacy in treating methamphetamine misuse [11]. However, subsequent randomized, double-blind, placebo-controlled trials of venlafaxine for cocaine or cannabis dependence reported inconsistent findings [12–14]. Clinical trials encounter limitations due to restrictive population designs that hinder its generalizability [15, 16]. In addition, outcomes in previous studies were focused on depression or craving [12–14]; therefore, evidence for the use of venlafaxine to reduce the risk of withdrawal in patients with nonopioid substance use has been limited.

According to real-world data, antidepressants were the most prevalent medications and were purchased by more than half of the patients with methamphetamine use disorder [16]. This study aimed to evaluate the association between the recent use of venlafaxine and the risk of substance withdrawal-related outcomes in patients with nonopioid substance use. We used a case-crossover design for this study to control for potential confounding factors, as venlafaxine may be prescribed for some comorbidities that can also be related to substance withdrawal. However, a clinician might also prescribe venlafaxine for individuals experiencing withdrawal symptoms from substance use, potentially leading to reverse causality (i.e., protopathic bias) in our analyses [17]. To address this issue, we conducted a case-case-time-control study that integrates two self-controlled analyses: a case-crossover analysis and a control-crossover analysis involving future cases [18].

## Methods

### Data source

The data for this study were obtained from four national databases in Taiwan (Supplementary Fig. 1). The first is Taiwan’s National Health Insurance Research Database (NHIRD), which is an administrative claims database associated with Taiwan’s National Health Insurance program and covers 99.9% of Taiwan’s population (approximately 23 million people). Details of the database have been described elsewhere[19]. Briefly, the database contains comprehensive records of diagnoses, drug prescriptions, procedures, and use in different health care settings[20]. The second is the National Cause of Death Registry, which contains information about the dates and causes of death. The data linkage between the NHIRD and the National Cause of Death Registry were highly complete [18].

The third is the Taiwan Illicit Drug Issue Database (TIDID), which is an interagency database on individuals with a history of illicit substance use in Taiwan [21]. The TIDID contains various data, including date of birth, sex, date of arrest, and categories of illicit substance use verified by urine tests. However, the data are unstructured, and nearly half of the records have missing values (250,000 out of 500,000 records). Therefore, we used the fourth national database, the Administrative Penalty System for Schedules III/IV Substances (APS) [22], to assist in identifying illicit substance use records. Although the APS has fewer records, the data quality is higher, with more structured data and fewer missing values (5,000 out of 17,000 records).

All the information of the study subjects underwent a deidentification process using a scrambling procedure [23]. We used complete datasets from the TIDID and APS databases and merged cases from both databases. We then linked the combined database to the NHIRD to obtain information about diagnoses, drug prescriptions, and dates of withdrawal events. Additionally, we linked the database to the National Cause of Death Registry to obtain information on the date of death.

These four national databases are maintained by the Health and Welfare Data Science Centre, which is a national agency tasked with protecting the privacy of subjects and providing scientists with access to databases for study purposes. The study was approved by the Institutional Review Board for the Protection of Human Subjects at Antai Medical Care Corporation (approval number: 22-105-A).

### Study population

**Fig. 1:**
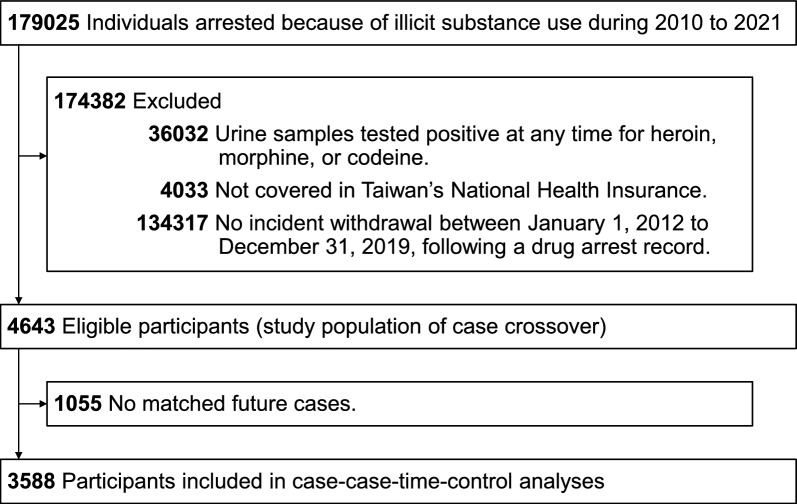
Flowchart of the study population assembly

We identified individuals with a history of illicit substance use from 2010 to 2021 from the TIDID and APS (*n* = 179,025). The first arrest date was defined as the entry date. We excluded those whose urine samples tested positive at any time for heroin, morphine, or codeine to focus on individuals who use nonopioid substances (Fig. 1). After linking eligible subjects from the TIDID to the NHIRD, we identified patients with incident withdrawal between 1 January 2012 and 31 December 2019 after the entry date (Fig. 1). The two-year periods before 2012 and after 2019 were used to ascertain whether patients had used opioids. The withdrawal events were identified based on diagnoses using the international classification of diseases, ninth and tenth revisions, clinical modification (ICD-9-CM code 292.0 or ICD-10-CM codes F1423, F1493, F1523, F1593). To investigate all-cause mortality, we identified patients who died based on the National Cause of Death Registry. We selected all-cause mortality as a primary outcome for two main reasons. First, within the context of substance use disorders, premature death is the most severe and definitive adverse outcome. Second, all-cause mortality is an unambiguous and reliably ascertained event, minimizing the risk of ascertainment bias [18].

### Study design

**Fig. 2:**
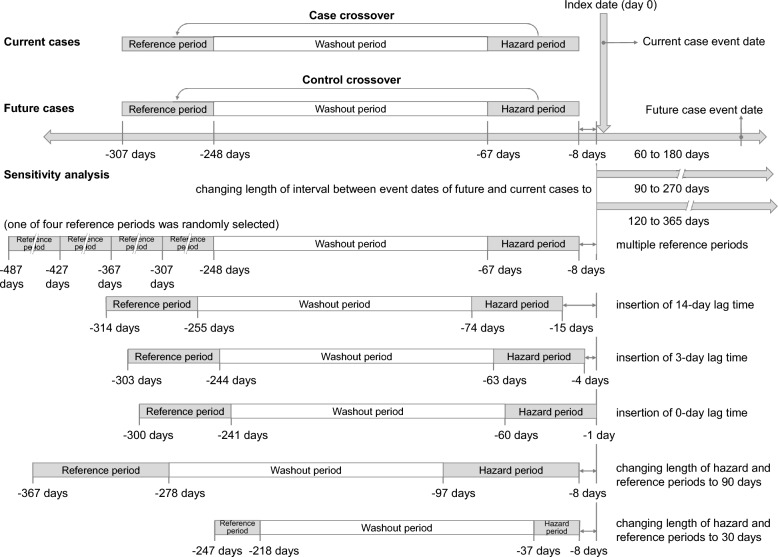
Schematic representation of the main analysis and sensitivity analyses

We used a case-case-time-control design in this study, which included two self-controlled analyses incorporating a case-crossover design and a control-crossover design with future cases [17, 18]. The case-crossover design eliminated time-invariant confounders through within-individual comparisons [24]. These participants act as their own control; therefore, time-constant covariates varying between individuals are controlled. Control-crossover analysis was performed to adjust for time trends in medication use and to address the likely protopathic bias by including future cases [17, 25]. Employing future cases not only addresses protopathic bias but also provides a comparison group more closely matched to current cases than using non-case subjects [17]. We matched current cases to future cases one-to-one by age and sex. Specifically, for future cases, we selected patients whose event dates were within 60–180 days after the event dates of the matched current cases [26]. Event dates were defined as the first date of events. We defined the index dates as the event dates of the current cases and assigned the same index dates for the future cases. A seven-day lag period was applied between the index date and the hazard period to mitigate the risk of protopathic bias. We divided the 307 days preceding the index dates into several 60-day intervals, including a hazard period (days − 8 to − 67), a washout period (days − 68 to − 247), and a reference period (between days − 248 and − 307). The selection of 60-day intervals was based on a previous clinical trial [27] and clinical practices in Taiwan. Figure 2 presents the case-case-time-control design and details of the time windows. Additionally, we selected mirtazapine as a control to perform a negative control analysis. In contrast to venlafaxine, mirtazapine inhibits histamine-1 receptors, resulting in prominent sedative effects and increased appetite [28]. Mirtazapine was selected because mirtazapine is also used to manage psychiatric disorders; therefore, patients may share similar comorbidities with those exposed to venlafaxine but without an effect on withdrawal from substances [7]. We expected that if the observed association with venlafaxine were driven by unmeasured confounding shared by antidepressant users, we would observe a similar association with mirtazapine.

### Statistical analysis and covariates

We defined the baseline period as one year prior to the index date during which data on comorbidities were collected (Table 1 and Supplementary Table S1). To evaluate the association between events and venlafaxine (ATC code: N06AX16) or mirtazapine (ATC code: N06AX11), we used conditional logistic regression to estimate the odds ratios with 95% confidence intervals, comparing the hazard period with the reference period. We calculated case-case-time-control odds ratios as the odds ratios from the case-crossover analysis divided by the odds ratios from the control-crossover analysis. Details of the 95% confidence interval calculation have been described elsewhere [18].

**Table 1:** Characteristics in baseline periods of eligible participants and current cases in the main analysis

Characteristics	Eligible participants (*n* = 4,643)	Current cases (*n* = 3,588)
Age (years), mean (SD)	39.5 (8.7)	39.3 (8.1)
Female, *n* (%)	738 (15.9)	484 (13.5)
*Insurance premium level, n (%)*		
< 28.8 K	4,074 (88.3)	3,128 (87.7)
28.8–45.8 K	185 (4.0)	146 (4.1)
> 45.8 K	35 (0.8)	27 (0.8)
Missing data	320 (6.9)	264 (7.4)
*Substance use, n (%)*		
Methamphetamine	2,583 (55.6)	1,973 (55)
Ketamine	531 (11.4)	387 (10.8)
Benzodiazepines	165 (3.6)	127 (3.5)
Ecstasy	93 (2.0)	66 (1.8)
New psychoactive substances^a^	70 (1.5)	49 (1.4)
Cannabis	42 (0.9)	32 (0.9)
Cocaine	26 (0.6)	23 (0.6)
Lysergic acid diethylamide	23 (0.5)	20 (0.6)
Multiple substances	577 (12.4)	425 (11.8)
Missing data	1,084 (23.3)	836 (23.3)
*Comorbidities, n (%)*		
Alcohol use disorder^b^	125 (2.7)	99 (2.8)
Anxiety disorder	1,275 (27.5)	970 (27.0)
Depressive disorder	1,013 (21.8)	775 (21.6)
Bipolar disorder	184 (4.0)	139 (3.9)
Schizophrenia	167 (3.6)	122 (3.4)
Chronic obstructive pulmonary disease	110 (2.4)	81 (2.3)
Diabetes	195 (4.2)	127 (3.5)
Hypertension	488 (10.5)	354 (9.9)
Hyperlipidemia	129 (2.8)	89 (2.5)
Heart failure	32 (0.7)	26 (0.7)
Epilepsy	64 (1.4)	50 (1.4)
Migraine	41 (0.9)	29 (0.8)
Parkinsonism	17 (0.4)	14 (0.4)
Osteoporosis	6 (0.1)	5 (0.1)

^a^New psychoactive substances include mephedrone, methylone, ethylone, chloroethcathinone, chloromethcathinone, chlorodimethylcathinone, ethylpentylone, butylone, butylone, methylpentedrone, methcathinone, and fluorodeschloroketamine

^b^The comorbidity of alcohol use disorder was based on the International Classification of Disease (ICD) code and therefore may be underdiagnosed

### Sensitivity analyses

We conducted a series of sensitivity analyses to assess the robustness of our findings (Fig. 2). Because there was no consensus on the length of the lag time, hazard, reference, or future case periods, we examined the assumptions of the lengths of the time periods for each time window. To assess the potential impact of protopathic bias, where patients with early symptoms of withdrawal may have a prescription before the event date, we changed the length of the lag time period from 7 to 14 days, 3 days and 0 day, respectively. We expected that if the association were driven by protopathic bias, the effect would attenuate with shorter lag times. We also changed the length of the hazard periods and reference periods from 60 to 90 days and 30 days, respectively. Furthermore, we redefined future cases by selecting patients whose events occurred between 90 and 270 days, and between 120 and 365 days after the event dates of the current cases. To account for the possibility that patients with opioid use disorder might lack arrest records in the TIDID [29], we conducted a sensitivity analysis by excluding patients with a diagnosis of opioid use disorder in the NHIRD (ICD-9-CM code 304.0, 304.7, 305.5, or ICD-10-CM codes F11.0 to F11.29). Because nonopioid substances may encompass different categories of illicit substances, we conducted a restrictive analysis by including only patients with stimulant use (i.e., methamphetamine or cocaine). To address potential time-varying confounding, such as a major depressive episode, could both influence antidepressant prescribing and withdrawal risk, we conducted a stratified case-crossover analysis. Specifically, we controlled for time-varying depression by using ICD-coded diagnoses in the week preceding the index date and estimating the effect of venlafaxine only within time windows concordant for depressive status (i.e., depression present in both case and referent periods or absent in both). This analysis assumes that if the association were driven primarily by the time-varying severity of depression, the effect would attenuate when comparisons are restricted to the periods of similar depressive status. To evaluate robustness to the reference-window specification, we constructed four consecutive 60-day reference periods and, for each case event, randomly selected one as the referent window.

We also performed a crude case-crossover analysis without adjustment for time trends among all eligible patients. To investigate the population-level time trends, we also conducted noncase control crossover analysis consisting of patients without an event between 2012 and 2019 (i.e., case-time-control analysis)[30]. We randomly matched controls to cases one-to-one based on the same index date and disease risk score [31] and repeated the analysis using the noncase group.

## Results

### Baseline characteristics of the study population

Of the 4,643 initially identified study patients, 3,588 current cases were included in the crossover analyses following one-to-one matching with future cases (Fig. 1). Table 1 outlines the baseline characteristics of the study population, including matched current and future cases. The average age of the study population was 39.5 years (standard deviation 8.7), with 84.1% being men and 88.3% having low income. The most common comorbidity was anxiety disorder (27.5%), followed by depressive disorder (21.8%). The most frequently recorded illicit substance was methamphetamine (55.6%). The baseline characteristics of the future cases were comparable to those of the current cases (Supplementary Table S2).

### Associations between recent venlafaxine use and withdrawal events

Table 2 provides details on the use of venlafaxine and mirtazapine during the hazard and reference periods. Fewer participants were prescribed venlafaxine during hazard periods than during reference periods. For instance, 18 patients in the current cases had received venlafaxine during the hazard period but not during the reference period, while 20 patients in the current cases exhibited the opposite pattern. In the analysis of recent venlafaxine use, the odds ratios were 0.90 (95% confidence interval 0.48 to 1.70) in the case-crossover analysis and 2.56 (1.18 to 5.52) in the control-crossover analysis. This resulted in a case-case-time-control odds ratio of 0.35 (0.13 to 0.96; Table 2). Conversely, for recent use of mirtazapine, the odds ratio was 1.07 (0.75 to 1.52) in the case-crossover analysis and 1.28 (0.90 to 1.82) in the control-crossover analysis, yielding a case-case-time-control odds ratio of 0.84 (0.51 to 1.38; Table 2).

**Table 2:** Results of odds ratio in case-case-time-control analysis

	Exposed only in hazard period	Exposed only in referent period	Exposed in both periods	Nonexposed in both periods	Odds ratio (95% CI)
*Venlafaxine*					
*Withdrawal from substances*					
Case-crossover	18	20	13	3,537	0.90 (0.48 to 1.70)
Control-crossover	23	9	9	3,547	2.56 (1.18 to 5.52)
Case-case-time-control	NA	NA	NA	NA	0.35 (0.13 to 0.96)
*Death*					
Case-crossover	46	27	23	7,208	1.70 (1.06 to 2.74)
Control-crossover	41	26	22	7,215	1.58 (0.97 to 2.58)
Case-case-time-control	NA	NA	NA	NA	1.08 (0.55 to 2.14)
*Mirtazapine*					
*Withdrawal from substances*					
Case-crossover	63	59	46	3,420	1.07 (0.75 to 1.52)
Control-crossover	69	54	41	3,424	1.28 (0.90 to 1.82)
Case-case-time-control	NA	NA	NA	NA	0.84 (0.51 to 1.38)
*Death*					
Case-crossover	212	120	159	6,813	1.77 (1.41 to 2.21)
Control-crossover	163	121	160	6,860	1.35 (1.07 to 1.70)
Case-case-time-control	NA	NA	NA	NA	1.31 (0.95 to 1.81)

CI, confidence interval; NA, not applicable

### All-cause mortality

Of the 8,601 initially identified study participants, we included 7,304 current cases in the crossover analyses following one-to-one matching with future cases (Supplementary Table S3). We observed no association between the recent use of venlafaxine and all-cause mortality, with a case-case-time-control odds ratio of 1.08 (0.55 to 2.14; Table 2). Similar results were observed for mirtazapine, with an odds ratio of 1.31 (0.95 to 1.81; Table 2).

### Sensitivity analyses

Table 3 presents the results of the sensitivity analyses, which included different lag time analyses, different hazard lengths and reference periods, different intervals between event dates of future cases and current cases, using multiple reference periods, adjusting for time-varying depressive disorder, and a restrictive analysis that included only patients with stimulant use. We interpret these results independently, recognizing that each analysis targets a distinct source of bias under differing identifying assumptions. When we inserted a lag time of 3 or 0 days before the index date, the odds ratios remained similar, but we observed wider 95% confidence intervals (Table 3). Using the case-time-control design for time trend adjustment, the results were comparable to those of the case-case-time-control analysis (odds ratio 0.60, 0.25 to 1.44).

**Table 3:** Sensitivity analyses

Analyses	Withdrawal from substances, odds ratio (95% confidence interval)	Death, odds ratio (95% confidence interval)
Main analysis	0.35 (0.13 to 0.96)	0.75 (0.39 to 1.45)
*Change the lag time from 7 days to*		
14 days	0.39 (0.17 to 0.88)	1.17 (0.77 to 1.75)
3 days	0.36 (0.13 to 1.02)	0.97 (0.49 to 1.92)
0 day	0.36 (0.13 to 1.01)	0.97 (0.49 to 1.92)
*Change the hazard and reference periods from 60 days to*		
90 days	0.48 (0.19 to 1.21)	0.70 (0.36 to 1.36)
30 days	0.66 (0.23 to 1.85)	0.72 (0.35 to 1.49)
*Change the Length of interval between event dates of future cases and current cases from 60–180 days to*		
90–270 days	0.62 (0.22 to 1.72)	0.74 (0.36 to 1.53)
120–365 days	0.69 (0.31 to 1.53)	0.75 (0.50 to 1.14)
Exclude patients with a diagnosis of opioid use disorder	0.40 (0.17 to 0.96)	0.89 (0.47 to 1.69)
Include patients with stimulants use only	0.49 (0.14 to 1.69)	1.13 (0.38 to 3.56)
Use multiple reference periods	0.65 (0.33 to 1.29)	0.88 (0.61 to 1.25)
Adjust for time-varying depressive disorder	0.46 (0.10 to 2.12)	1.48 (0.63 to 3.44)
Crude case-crossover analysis (*n* = 4,643)	0.89 (0.51 to 1.55)	1.15 (0.75 to 1.74)
Case-time-control analysis (*n* = 4,259)	0.60 (0.25 to 1.44)	1.72 (0.82 to 3.65)

## Discussion

In this nationwide, population-based study, we observed that venlafaxine use was associated with a reduced risk of experiencing withdrawal symptoms from nonopioid substances during the hazard period compared with the reference period. This result was based on correction for the potential time trends of increasing venlafaxine use at the population level, as estimated by the control crossover analysis consisting of noncases, and additional correction for the individual-level time trends estimated by the control crossover analysis consisting of future cases. Moreover, exposure to mirtazapine, another antidepressant used as a negative control, was not associated with withdrawal from substances. These results strengthened the reliability of the findings from the analysis of venlafaxine. Currently, there is no medication available for nonopioid substance use to reduce withdrawal from substances. Our findings may provide a good foundation for exploring venlafaxine as an option for those with nonopioid substance use.

Case-crossover analysis is an efficient tool that can evaluate transient exposure and acute outcomes, conditioned on time-constant confounders by using self-controlled designs. However, the method is sensitive to time trends. For example, from the control crossover analysis consisting of noncases, we found a time trend resulting in the hazard window having a 1.47-fold greater likelihood of exposure to the medication compared to the reference window (Supplementary Table S4). This may suggest that venlafaxine has become more commonly used over time, either for individuals experiencing withdrawal symptoms from substance use or among all populations in clinical practice. Without adjusting for such time trends, the benefits of venlafaxine in reducing withdrawal from substances could not be accurately observed. Moreover, the control crossover analysis consisting of future cases may reflect a potential combination of effects from both population-level and individual-level time trends. The odds ratio (2.56) from the future case–control crossover was greater than the odds ratio (1.47) from the noncase–control crossover, suggesting that an individual-level time trend could not be omitted (Supplementary Table S4). A likely explanation could be that clinicians may prescribe venlafaxine for the early manifestation of withdrawal from substances that has not yet been diagnosed, leading to potential protopathic bias (or reverse causation). The case-case-time-control analysis, which incorporates both a case-crossover analysis and a control crossover consisting of future cases, helped to correct this bias in our study. These analyses may also reveal the possible reason why these benefits were not observed in previous studies [16].

The inconsistent findings in previous clinical trials may also be attributed to a high dropout rate (33%[13] ~ 67%[14]), as venlafaxine was associated with more adverse events [32]. Furthermore, adverse events can lead to the acute discontinuation of venlafaxine, potentially leading to withdrawal [33]. This discontinuation may also contribute to an increase in cannabis use [13, 34] and was associated with worse abstinence outcomes [32]. In addition, the potential treatment effects of venlafaxine may be short, considering that measures of depression improved more rapidly with venlafaxine than with placebo, but these differences disappeared after 6–8 weeks [14]. All these factors undermine the potential therapeutic effects of venlafaxine within the constraints of experimental designs in clinical trials. Future studies could focus on accounting for the heterogeneous vulnerability to adverse events, focusing on particular subgroups that may derive benefits from venlafaxine, or developing strategies to enhance adherence in participants receiving venlafaxine. Additionally, the most frequently recorded illicit substance in our sample was methamphetamine, while previous clinical trials have focused mainly on cocaine [12, 14] or cannabis [13]. Moreover, our cohort consisted of patients encountered in everyday practice who may be ineligible for clinical trials. For example, a total of 88% of patients were in the low-income category, whereas this proportion was only 40% in clinical trials [14]. Clinical trials frequently exclude patients with co-occurring psychiatric conditions [12] or those at risk for suicide [13, 14]. Therefore, our study may have greater generalizability.

The potential therapeutic effects of venlafaxine may stem from increased norepinephrine in the synapse. Our rationale was supported by bupropion, which shares features of norepinephrine uptake inhibitors, in a nationwide, matched retrospective cohort study for cocaine use disorder [35] and a meta-analysis of placebo-controlled randomized clinical trials for methamphetamine use disorder [36]. It has been suggested that bupropion directly targets genetic pathways, including serotonergic synapses, and may, therefore, potentially modulate serotonin transport to facilitate more manageable or tolerable withdrawal from stimulants and remission [35]. Additionally, venlafaxine and bupropion potentially alleviate the symptoms of withdrawal from stimulants [37] in patients who commonly experience sedative effects and increased appetite [38]. These mechanisms may demonstrate the benefit of venlafaxine in withdrawal from substances for those with nonopioid substance use beyond its function as an antidepressant.

The negative effect of mirtazapine on reducing withdrawal is consistent with results from a previous study [7] and further strengthens the reliability of the positive findings for venlafaxine. Other antidepressants, such as selective serotonin reuptake inhibitors, are not recommended for individuals who use cocaine [37] or methamphetamine [39]. Nevertheless, antidepressants remain one of the most common choices for individuals who use substances, primarily due to their frequent comorbidity with depressive and anxiety disorders [40, 41], as well as high suicide rates [42]. Given that few antidepressants have proven to be effective in withdrawal from substances, the positive outcome we observed with venlafaxine becomes even more valuable. Clinicians may consider the use of venlafaxine to reduce patient experiencing withdrawal symptoms from nonopioid substances use.

## Limitations

We acknowledge some limitations that may introduce bias. The study patients were those who had been arrested for illicit substance use, which may not fully reflect real-world situations where individuals with hidden drug use may constitute more than 50%, and potentially up to 90%, of the total cases of drug misuse [43]. Nonetheless, even comprehensive national surveys encounter the problem of invisible cases [44]. Our study may have greater generalizability than clinical trials and better reflect the reality in clinical practice, given that a joint legal-medical treatment program (with addiction treatment as an alternative to imprisonment) comprises the majority of addiction outpatient cases in Taiwan [45]. The inability to assess adherence to venlafaxine prevented us from confirming whether patients received venlafaxine. However, this bias did not affect our conclusion because we could expect the effects of medication adherence to be consistent across the hazard and reference periods, resulting in a similar impact on the estimates. Furthermore, we did not include time-varying confounders in in the primary analyses [18]. Nevertheless, we performed a negative control analysis with mirtazapine as a control exposure and may have examined some time-varying confounders, such as major depressive episodes [46].

We acknowledge limitations of ICD-coded withdrawal events related to validation and of all-cause mortality related to cause specificity, warranting cautious interpretation. However, we selected these outcomes due to their significant clinical relevance and objectivity as hard endpoints. Prior validation supports the general reliability of NHIRD diagnoses [19]. More importantly, Taiwan's National Health Insurance only reimburses treatment for withdrawal, strongly incentivizing clinicians to use withdrawal-specific ICD codes, making them strong indicators of clinically significant events. In addition, there was no consensus on the length of hazard periods. We defined hazard periods based on the therapeutic profile of venlafaxine [27] and tested robustness through sensitivity analyses using alternative hazard-period lengths. While statistical power was sufficient to detect an effect in the primary analysis, it was limited in some sensitivity analyses given the limited sample size. Notably, our study utilizes a nationwide dataset linking several unique databases, making our sample size highly representative. Real-world data do not permit the application of all information available in clinical trials because only diagnoses are available, and some symptoms are often underreported in diagnostic data. Nonetheless, the self-controlled analysis inherently eliminated time-constant confounders.

## Conclusions

We found that the use of venlafaxine was associated with a reduced risk of withdrawal from substances when the time trends of medication were corrected through a control analysis consisting of future cases. These findings suggested that venlafaxine may be considered for use in clinical practice to reduce withdrawal for those with nonopioid substance use.

## Supplementary Information

Additional file1 (DOCX 1288 kb)

## Data Availability

The authors remotely accessed the data from the data centre of the Ministry of Health and Welfare in Taiwan. Researchers interested in accessing this dataset could submit a formal application to the Taiwan Ministry of Health and Welfare to request access (No 488, Sect. 6, Zhongxiao E Rd, Nangang District, Taipei 115, Taiwan; website: https://dep.mohw.gov.tw/DOS/cp-2516-59203-113.html). No additional data available due to confidentiality issues and the sensitive nature of forensic autopsy data.
