# Supplementary material for: Association between venlafaxine use and the risk of withdrawal from nonopioid substances: a nationwide, population-based study
Source: Harm Reduct J. 2026 Mar 2;23:71. doi: 10.1186/s12954-026-01427-9 (PMC13059312; doi:10.1186/s12954-026-01427-9)
Supplement: Supplementary file 1 — Additional file1 (DOCX 1288 kb) [file 12954_2026_1427_MOESM1_ESM.docx]

**Supplementary Materials**

**Supplementary Figure S1.** Diagram of data linkage among four nationwide databases

**Supplementary Table S1.** Comorbidities collected for baseline characteristics and used for disease risk score matching

**Supplementary Table S2.** Characteristics in baseline periods of current cases and future cases in the main analysis

**Supplementary Table S3.** Characteristics in baseline periods of eligible participants, current cases and future cases for all-cause mortality

**Supplementary Table S4.** Results of the primary analysis and sensitivity analyses of the crude case-crossover and case-time-control analyses

**Supplementary Figure S1.** Diagram of data linkage among four nationwide databases


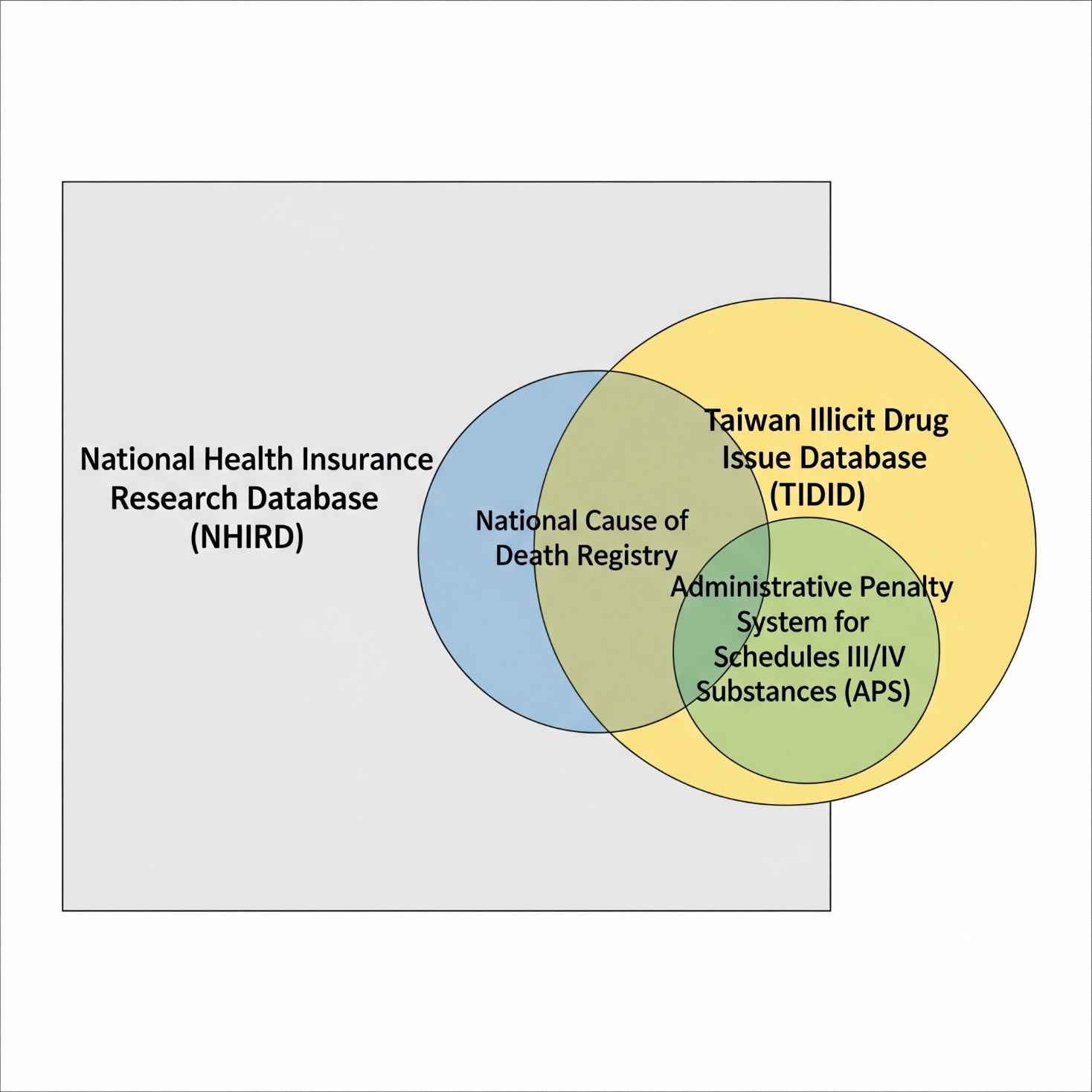


The diagram illustrates the relationships between the databases used. The National Health Insurance Research Database (NHIRD) serves as the foundational database, covering 23 million individuals. The National Cause of Death Registry is a subset of the NHIRD. The Taiwan Illicit Drug Issue Database (TIDID), covering 179,025 individuals, and its intersection with the NHIRD consists of 174,992 individuals. The Administrative Penalty System for Schedules III/IV Substances (APS), with 81,196 individuals, is fully contained within the TIDID, and its intersection with the NHIRD consists of 80,732 individuals. Data elements utilized:

From NHIRD: Diagnoses, drug prescriptions, and insurance premium level.

From National Cause of Death Registry: Dates of death.

From TIDID: Date of birth, sex, date of arrest, and categories of illicit substance use.

From APS: Date of arrest and categories of illicit substance use.

**Supplementary Table S1. Comorbidities collected for baseline characteristics and used for disease risk score matching**

| **Characteristics** | **ICD-9-CM** | **ICD-10-CM** |
| --- | --- | --- |
| Alcohol use disorder | 291, 3050 | F10 |
| Opioid use disorder | 304.0, 304.7, 305.5 | F11.0, F11.1, F11.2 |
| Anxiety disorder | 3000, 3002-3003, 3009 | F40, F41 |
| Depressive disorder | 2962, 2963, 29682, 3004, 311 | F32, F33, F341 |
| Bipolar disorder | 2960-2961, 2964-2967, 29680-81 | F30, F31 |
| Schizophrenia | 2950-2959 | F20, F21, F25 |
| Chronic obstructive pulmonary disease | 491, 492, 496 | J41-J44 |
| Diabetes | 2500-2509 | E10-E14 |
| Epilepsy | 3450-3459 | G40-G41 |
| Hypertension | 401-405 | I10-I16 |
| Hyperlipidemia | 272 | E78 |
| Heart failure | 428 | I50 |
| Migraine | 346 | G43 |
| Osteoporosis | 7330 | M80-M82 |
| Parkinsonism | 3320-3321 | G20, G21 |

A 3-digit ICD code can include more specific fourth and fifth digits. For example, the ICD-9-CM code “291” includes the wildcard code “291.xx”.

**Supplementary Table S2. Characteristics in baseline periods of current cases and future cases in the main analysis**

| Characteristics | Current cases (*n*=3 588) | Future cases (*n*=3 588) |
| --- | --- | --- |
| **Age (years), mean (SD)** | 39.3 (8.1) | 39.0 (8.2) |
| **Female, *n* (%)** | 484 (13.5) | 484 (13.5) |
| **Insurance premium level, *n* (%)** |  |  |
| < 28.8 K | 3 128 (87.7) | 3 143 (88.2) |
| 28.8–45.8 K | 146 (4.1) | 138 (3.9) |
| > 45.8 K | 27 (0.8) | 27 (0.8) |
| Missing data | 264 (7.4) | 255 (7.2) |
| **Drug records, *n* (%)** |  |  |
| Methamphetamine | 1 973 (55) | 2 042 (56.9) |
| Ketamine | 387 (10.8) | 418 (11.6) |
| Benzodiazepines | 127 (3.5) | 127 (3.5) |
| Ecstasy | 66 (1.8) | 75 (2.1) |
| New psychoactive substances ^a^ | 49 (1.4) | 56 (1.6) |
| Cannabis | 32 (0.9) | 33 (0.9) |
| Cocaine | 23 (0.6) | 20 (0.6) |
| Lysergic acid diethylamide | 20 (0.6) | 19 (0.5) |
| Multiple substances | 425 (11.8) | 452 (12.6) |
| Missing data | 836 (23.3) | 791 (22) |
| **Comorbidities, *n* (%)** |  |  |
| Alcohol use disorder ^b^ | 99 (2.8) | 93 (2.6) |
| Anxiety disorder | 970 (27.0) | 984 (27.4) |
| Depressive disorder | 775 (21.6) | 763 (21.3) |
| Bipolar disorder | 139 (3.9) | 136 (3.8) |
| Schizophrenia | 122 (3.4) | 122 (3.4) |
| Chronic obstructive pulmonary disease | 81 (2.3) | 79 (2.2) |
| Diabetes | 127 (3.5) | 142 (4.0) |
| Hypertension | 354 (9.9) | 381 (10.6) |
| Hyperlipidemia | 89 (2.5) | 92 (2.6) |
| Heart failure | 26 (0.7) | 21 (0.6) |
| Epilepsy | 50 (1.4) | 51 (1.4) |
| Migraine | 29 (0.8) | 27 (0.8) |
| Parkinsonism | 14 (0.4) | 14 (0.4) |
| Osteoporosis | 5 (0.1) | 4 (0.1) |

^a^ New psychoactive substances include mephedrone, methylone, ethylone, chloroethcathinone, chloromethcathinone, chlorodimethylcathinone, ethylpentylone, butylone, butylone, methylpentedrone, methcathinone, and fluorodeschloroketamine.

^b^ The comorbidity of alcohol use disorder was based on the International Classification of Disease (ICD) code and therefore may be underdiagnosed.

**Supplementary Table S3. Characteristics in baseline periods of eligible participants, current cases and future cases for all-cause mortality**

| Characteristics | Eligible participants (*n*=8 601) | Current cases  (*n*=7 304) | Future cases  (*n*=7 304) |
| --- | --- | --- | --- |
| **Age (years), mean (SD)** | 46.1 (10.6) | 46 (9.9) | 45.7 (10.1) |
| **Female, *n* (%)** | 778 (9) | 488 (6.7) | 488 (6.7) |
| **Insurance premium level, *n* (%)** |  |  |  |
| < 28.8 K | 7 656 (89.6) | 6 511 (89.7) | 6 470 (89.2) |
| 28.8–45.8 K | 392 (4.6) | 328 (4.5) | 346 (4.8) |
| > 45.8 K | 112 (1.3) | 87 (1.2) | 97 (1.3) |
| Missing data | 387 (4.5) | 336 (4.6) | 343 (4.7) |
| **Drug records, *n* (%)** |  |  |  |
| Methamphetamine | 3 787 (44) | 3 181 (43.6) | 3 282 (44.9) |
| Ketamine | 629 (7.3) | 492 (6.7) | 525 (7.2) |
| Benzodiazepines | 88 (1.0) | 67 (0.9) | 82 (1.1) |
| Ecstasy | 101 (1.2) | 76 (1) | 84 (1.2) |
| New psychoactive substances ^a^ | 16 (0.2) | 13 (0.2) | 12 (0.2) |
| Cannabis | 16 (0.2) | 14 (0.2) | 15 (0.2) |
| Cocaine | 9 (0.1) | 7 (0.1) | 9 (0.1) |
| Lysergic acid diethylamide | 6 (0.1) | 3 (0) | 3 (0) |
| Multiple substances | 398 (4.6) | 307 (4.2) | 341 (4.7) |
| Missing data | 3 204 (37.3) | 2 745 (37.6) | 2 651 (36.3) |
| **Comorbidities, *n* (%)** |  |  |  |
| Alcohol use disorder ^b^ | 571 (6.6) | 484 (6.6) | 497 (6.8) |
| Anxiety disorder | 1 812 (21.1) | 1 547 (21.2) | 1 553 (21.3) |
| Depressive disorder | 1 763 (20.5) | 1 486 (20.3) | 1 474 (20.2) |
| Bipolar disorder | 340 (4.0) | 251 (3.4) | 283 (3.9) |
| Schizophrenia | 382 (4.4) | 328 (4.5) | 308 (4.2) |
| Chronic obstructive pulmonary disease | 652 (7.6) | 527 (7.2) | 537 (7.4) |
| Diabetes | 1 234 (14.3) | 1 029 (14.1) | 1 039 (14.2) |
| Hypertension | 2 037 (23.7) | 1 692 (23.2) | 1 751 (24) |
| Hyperlipidemia | 603 (7.0) | 519 (7.1) | 516 (7.1) |
| Heart failure | 561 (6.5) | 469 (6.4) | 464 (6.4) |
| Epilepsy | 305 (3.5) | 259 (3.5) | 260 (3.6) |
| Migraine | 77 (0.9) | 63 (0.9) | 63 (0.9) |
| Parkinsonism | 77 (0.9) | 61 (0.8) | 64 (0.9) |
| Osteoporosis | 40 (0.5) | 29 (0.4) | 31 (0.4) |

^a^ New psychoactive substances include mephedrone, methylone, ethylone, chloroethcathinone, chloromethcathinone, chlorodimethylcathinone, ethylpentylone, butylone, butylone, methylpentedrone, methcathinone, and fluorodeschloroketamine.

^b^ The comorbidity of alcohol use disorder was based on the International Classification of Disease (ICD) code and therefore may be underdiagnosed.

**Supplementary Table S4. Results of the primary analysis and sensitivity analyses of the crude case-crossover and case-time-control analyses**

| Odds ratio (95% CI) | **Primary analysis** | **Sensitivity analyses** | |
| --- | --- | --- | --- |
|  | Case-case-time-control (CCTC) analysis | Case-time-control (CTC) analysis | Crude case-crossover |
| **Substance withdrawal** |  |  |  |
| Case-crossover | 0.90 (0.48 to 1.70) | 0.88 (0.49 to 1.57) | 0.89 (0.51 to 1.55) |
| Control-crossover | 2.56 (1.18 to 5.52) | 1.47 (0.76 to 2.83) | - |
| Case/control ratio | 0.35 (0.13 to 0.96) | 0.60 (0.25 to 1.44) | - |
| **Death** |  |  |  |
| Case-crossover | 1.70 (1.06 to 2.74) | 1.45 (0.90 to 2.33) | 1.15 (0.75 to 1.74) |
| Control-crossover | 1.58 (0.97 to 2.58) | 0.84 (0.47 to 1.50) | - |
| Case/control ratio | 1.08 (0.55 to 2.14) | 1.72 (0.82 to 3.65) | - |

CI=confidence interval.
